# Investigation of 1,4-Substituted 1,2,3-Triazole Derivatives as Antiarrhythmics: Synthesis, Structure, and Properties

**DOI:** 10.3390/ph15121443

**Published:** 2022-11-22

**Authors:** Elena O. Shestakova, Sergey G. Il’yasov, Irina A. Shchurova, Vera S. Glukhacheva, Dmitri S. Il’yasov, Egor E. Zhukov, Arkady O. Bryzgalov, Tatiana G. Tolstikova, Yuri V. Gatilov

**Affiliations:** 1 Institute for Problems of Chemical and Energetic Technologies, Siberian Branch of the Russian Academy of Sciences (IPCET SB RAS), 659322 Biysk, Russia; 2 Novosibirsk Institute of Organic Chemistry, Siberian Branch of the Russian Academy of Sciences (NIOCh SB RAS), 630090 Novosibirsk, Russia

**Keywords:** azide-alkyne cycloaddition (CuAAC), azidoalkyl nitramines, 1,3-diazido-2-nitro-2-azapropane (DANP), antiarrhythmic

## Abstract

Here, we investigated the reaction of 1,3-dipolar cycloaddition of 1,3-diazido-2-nitro-2- azapropane (DANP) to propargyl alcohol over a copper-based catalyst and identified the optimum reaction conditions that enable the synthesis of 2-nitro-1,3-bis(4,4′-dihydroxymethyl)-1,2,3-triazolyl-2-azapropane (**1**) in more than 84% yield. The reaction between DANP, 1,5-diazido-3-nitrazapentane, and phenylacetylene produced the respective 1,2,3-triazole derivatives in 83% and 71% yields, respectively. The structures of the resultant compounds were validated by infrared and NMR spectroscopies and elemental analysis. The structure of **1** was proved by single-crystal X-ray diffraction. This study demonstrated that **1** exhibits a dose-dependent antiarrhythmic activity towards calcium-chloride-induced arrhythmia and refers to Class III: moderately hazardous substances.

## 1. Introduction

1,2,3-Triazole derivatives hold an important place in organic chemistry because they are employed as intermediate products in the organic synthesis [1,2,3], are attractive as high-energy materials [1,4], and exhibit a wide range of biological activities (analgesic, sedative, antimicrobial, antiviral, anti-HIV, antifungal, antitumor, etc.) [5,6,7,8]. In addition, 1,2,3-triazole derivatives were shown to be able to affect and block the L-type calcium channels, which is of interest in itself for the study of an antiarrhythmic effect comparable with the common antiarrhythmic drugs blocking the calcium channels [9].

L-type calcium channels, which were cloned first and remain the most studied, are found in the plasma membrane of skeletal muscle, myocardium, and smooth muscle, as well as neurons. Their activation occurs during membrane depolarization. These channels can remain open for a long time. Calcium channels of L-type are activated at high potentials on the membrane (over 10 mV), are characterized by high conductivity (25 pSm) and very slow kinetics of inactivation (t > 500 ms) in contrast to T-type channels, which are found in many excitable and nonexcitable (fibroblasts, B-lymphocytes) cells, and are activated by weak depolarization (potentials more positive than −70 mV), quickly and potentially dependent [9].

1,2,3-Triazoles are available compounds. The most general synthetic method for substituted 1,2,3-triazoles is the reaction of 1,3-dipolar cycloaddition between azides and alkynes [2]. The copper-catalyzed option of the azide-alkyne cycloaddition reaction (CuAAC) is regioselective and furnishes 1,4-disubstituted 1,2,3-triazoles as major products [10,11,12,13]. The mechanism of this reaction is multistage and has been studied in detail elsewhere [14,15].

The literature [1] describes the azide-alkyne cycloaddition of 1,5-diazido-3-nitrazapentane (DIANP) to propargyl alcohol over a copper catalyst to synthesize 3-nitro-1,5-bis(4,4′-dihydroxymethyl)-1,2,3-triazolyl-3-azapentane (**2**), starting from which the respective 1,2,3-triazole diazido derivative was prepared and characterized by physicochemical methods.

**Figure 1:**
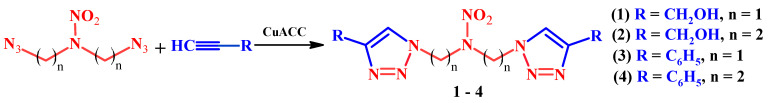
A general synthetic protocol for 1,4-substituted 1,2,3-triazoles.

We previously developed a process for the synthesis of a linear azidomethyl nitramine, 1,3-diazido-2-nitro-2-azapropane (DANP) [16], from the corresponding dichloro derivative which is currently viewed as a building block for the preparation of caged compounds [17,18,19,20,21,22]. In this regard, it is an interesting research objective to expand the limits of potential application of DANP through its chemical transformation, in particular, through its azide-alkyne cycloaddition to terminal alkynes to yield 1,4-substituted 1,2,3-triazoles and to examine their toxicology and pharmacology (Figure 1). 

To examine the cyclization reaction of DANP, we chose propargyl alcohol and phenylacetylene, which are widely used for that purpose. It is known that one of the most common options to carry out this reaction is the reaction in an aqueous medium over a copper sulfate/sodium ascorbate catalytic system [4,8,10,11,12]. However, as applied to our compounds, this method is not suitable because the starting reactants are insoluble in water, while the final product is well-soluble and hinders their isolation. Moreover, being soluble in the reaction mixture, copper sulfate makes its regeneration impossible and hinders the isolation of products as well. Therefore, the present study aimed to explore if removable heterogeneous catalysts, such as copper wire, copper powder, and copper nanooxide (n-CuO), can be used in different organic solvents and if thermal cyclization can be carried out without catalysts. Using the model reaction of azide-alkyne cycloaddition of DANP to propargyl alcohol, we looked into the effects of the catalyst, solvent nature, and molar ratio of the reactants on the reaction length, product yield, and quality.

## 2. Results and Discussion

### 2.1. Synthesis

The effects of copper-based catalysts (copper wire, powdered copper, and copper oxide) on the reaction length of azide-alkyne cycloaddition of DANP to propargyl alcohol, yield, and quality of compound **1** were evaluated under the following conditions: a 1:2:2 molar ratio of the reactants (DANP:propargyl alcohol:catalyst), tert-butyl alcohol (12% DANP solution) as the solvent, and a 35 °C reaction temperature. The reaction was performed until the starting azide disappeared from the reaction mixture and monitored by thin-layer chromatography (3.5:1.5 benzene:methanol as the eluent). The evaluation results are summarized in Table 1. 

As expected, an increase in the specific surface of the catalyst reduced the reaction time and enhanced the yield of compound **1**, but because there were technological difficulties isolating the target product when copper nanooxide was used as the reaction catalyst, it was more expedient to perform further studies with copper powder (entry 3). 

The reaction of cycloaddition of DANP to propargyl alcohol proceeded in a heterogeneous medium to furnish compound **1** as a white sediment mixed with the catalyst upon reaction completion. To separate the catalyst, acetonitrile was added to the mixture and heated until the sediment was fully dissolved, the catalyst was removed, the stock solution was cooled, and crystalline product **1** was isolated. This approach was further employed as a method to work up compound **1**. The temperature effect on the reaction time and yield of **1** was investigated using *t*-butanol (Table 2).

Table 2 shows that a rise in the reaction temperature led to a natural reduction in the reaction time but had no considerable impact on the yield of compound **1**. Despite the long reaction time, the reaction is more convenient to carry out at room temperature of 20–25 °C. 

Here, we examined if DANP could be cyclized with propargyl alcohol under thermal cyclization conditions with no catalyst added. The reaction was carried out in *t*-butanol and acetonitrile (because it was employed in the target product isolation) at 80 °C until azide disappeared from the reaction mixture (TLC monitoring). The reaction was very slow and nonselective. The reaction in acetonitrile was completed in 6 days to furnish four more impurities along with compound **1** (a 15.7% product content as per HPLC). The reaction in *t*-butanol was even slower and 29.05% of the starting azide was left, while the content of compound **1** was 5.3% and four impurities were also present. 

Since the solvent nature has an effect on the reaction length of CuAAC, we considered methanol and ethanol, acetone, methylene chloride, chloroform, tetrahydrofuran (THF), and toluene as the reaction medium, in addition to *t*-butanol and acetonitrile. The reaction was carried out at 20–25 °C. The study results regarding the effect of the above-listed solvents on the yield and quality of compound **1** are summarized in Table 3.

Among the solvents tested (Table 3), the highest yields of **1** were obtained in the chlorinated organic solvents; in addition, the crystalline product was isolated with minimum losses because of a lower solubility, as opposed to the alcohols and acetone. Technologically, methylene chloride is more preferable, as it is used to isolate DANP [16] and DIANP [26,27].

Further, we looked into how the molar ratio of azide:alkyne (propargyl alcohol) ranging from 1:2–1:2.4 influenced the reaction time and yield of compounds **1** and **2** in methylene chloride over a catalyst (copper powder) at a holding time of 20–25 °C. The results are outlined in Table 4.

It was found that a slight excess of alkyne should be used to enhance the yield of 1,4-substituted 1,2,3-triazoles **1** and **2**. Among the azide:alkyne molar ratios of 1:2, 1:2.2, and 1:2.4, the highest yield was achieved when the ratio was 1:2.2. The stepwise mechanism of the copper-catalyzed reaction of azide-alkyne cycloaddition proceeded through an intermediate formation of copper acetylenides. The reaction was likely to be very sensitive to the reactant ratio affecting the reaction length. It is seen from Table 4 that the stoichiometric ratio of azide:alkyne (1:2.0) was not sufficient; the reaction proceeded for quite a long time to generate a lot of impurities. A slight excess of 10% alkyne (1:2.2 ratio) considerably increased the reaction rate and was optimum. A higher excess of the alkyne (1:2.4 ratio) influenced the solvent properties by imparting the polarity to it, and slightly diminished the yield in spite of the process being accelerated, as is confirmed by the data in Table 3 by the example of the alcohols. 

In order to increase the purity of compound **1**, we considered the process of recrystallization as the purification method. The solubility of **1** was initially explored in different solvents, and the following solvents were chosen following the recrystallization results: acetonitrile, acetone, ethanol, and isopropyl alcohol (IPA). The recrystallization process was run as follows: compound **1** was dissolved in a minimum volume of the boiling solvent, the hot solution was filtered and cooled down, and the precipitated crystalline product **1** was isolated from the stock solution. An averaged sample of compound **1** with 94.4% purity was employed as the sample for purification. The purity of the samples of compound **1** was monitored by HPLC. The test results are summarized in Table 5.

It is seen from the data in Table 5 that the most efficient monosolvent for recrystallization of compound **1** is acetone (entry 2), allowing the purity to be enhanced from 94.4% to 99.2%. As compound **1** is well-soluble in water, we used mixed acetone/water (entry 6) in order to reduce the amount of acetone. Along with the high product quality, this considerably reduced the solvent usage and increased the product yield from recrystallization.

Thus, we consequently identified the optimum CuAAC reaction conditions of DANP with propargyl alcohol and proposed the purification method for compound **1** via recrystallization from mixed acetone/water, affording the target product with 99.9% purity.

Based on the findings, we synthesized a range of derivatives (**1**–**4**) by the reaction of azide-alkyne cycloaddition of DANP and DIANP to propargyl alcohol and phenylacetylene (Table 6).

As is seen from Table 6, the derivatives 2-nitro-1,3-bis(4,4′-diphenyl)-1,2,3-triazolyl-2-azapropane (**3**) and 3-nitro-1,5-bis(4,4′-diphenyl)-1,2,3-triazolyl-3-azapentane (**4**) were generated in quite high yields of 83% and 71%, respectively.

### 2.2. Infrared Spectroscopy

A comparative analysis of the IR spectra (Supplementary Materials) between the synthesized compounds **1**, **3**, **4,** and compound **2** reported in [1] demonstrated that the IR spectra have representative absorption bands of functional groups relating to compound **2**, that is: *ν* N–NO_2_ (1571–1578 cm^−1^, 1273–1284 cm^−1^); CH_2_ (2997–2850 cm^−1^, 1466–1444 cm^−1^), *ν* C–H (3098–3017 cm^−1^), *ν* N=N of 1,2,3-triazolyl groups (1417–1424 cm^−1^) and *ν* N–N of nitramine (1098–1064 cm^−1^) (Table 7).

**Figure 2:**
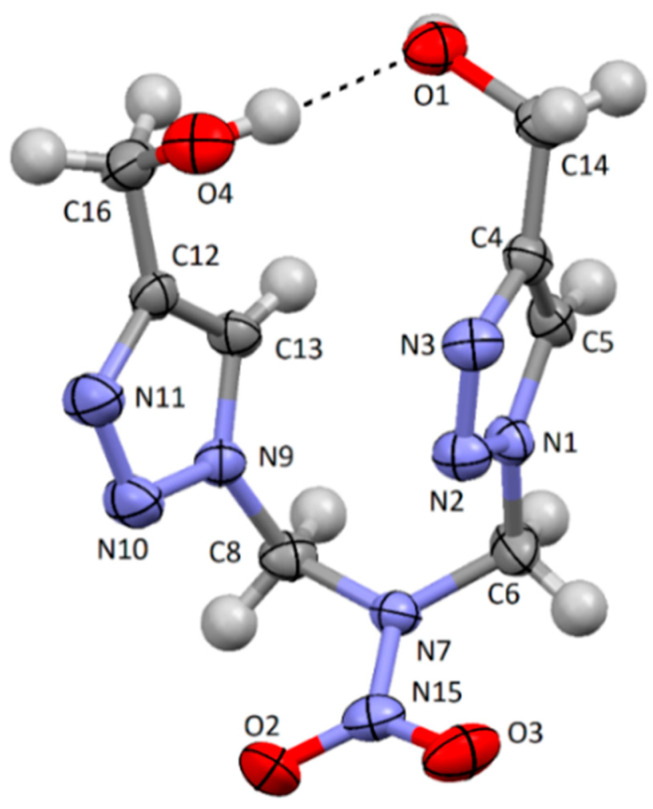
The molecular structure of compound **1** with 50% thermal ellipsoids.

It is seen from Table 7 that the IR spectrum of compound **1** has a strong absorption band at 3380 cm^−1^ which can be referred to the intramolecular coupling between H of the hydroxyl group and the adjacent O of the hydroxyl. This assumption was proved by single-crystal X-ray diffraction analysis (Figure 2). The existent band at 3300 cm^−1^ for compound **2** [1] should also be assigned to the intramolecular H-bond.

### 2.3. NMR Spectroscopy

The structures of the resultant compounds **1**–**4** were verified by ^1^H and ^13^C NMR spectroscopy. The ^1^H NMR spectra of the synthesized compounds **1**–**4** have a chemical shift of the H5 atom of the 1,2,3-triazole system as a singlet in the range of 8.00–8.75 ppm and other representative signals. The ^13^C NMR spectra show representative signals of C5 and C4 carbon atoms in the range of 122.46–127.49 and 139.27–148.64 ppm, which is in agreement with the data reported in [1] and proves the formation of 1,4-disubstituted 1,2,3-triazoles.

### 2.4. DSC and TGA

The thermal stability of compound **1**, as measured by DSC and TGA thermogravimetric analyses (Supplementary Materials), can be split into two stages: melting (endothermic effect) and decomposition (exothermic effect) of the sample (Table 8). For instance, the first stage in the DSC spectrum exhibits two endothermic peaks at 115.6 °C and 145.9 °C with a specific heat absorption of −7.6 J/g and −5.2 J/g, respectively, with no weight loss (TGA) until 224 °C. The appearance of two and more endothermic peaks in the DSC spectrum is characteristic of substances having polymorphic modifications. The second stage exhibits two exothermic peaks at 249.0 °C and 363.6 °C, with a specific heat release of 55.1 J/g and 29.3 J/g, respectively. That said, a two-step weight loss of 45.96% at the first decay step and 34.92% at the second decay step is observed. The undecomposed residue was estimated to be 19.12%, which is equivalent to the value in the DSC spectrum. The two-step (and more) decay of a substance is typical of secondary reactions of decay products of the major compound to generate new, more thermally stable compounds. 

### 2.5. X-ray Data for Compound ***1***

The structure of compound **1** was definitely verified by X-ray crystallography. The U-like conformation of the molecule was formed by the intramolecular H-bond O4–H…O1 with H…O 1.96(2) and O…O 2.828(2) Å distances and O–H…O 177(2)° angle (Figure 2).

X-ray crystallographic data were obtained on a Kappa Apex II diffractometer (Bruker, Billerica, MA, USA) with a CCD detector using graphite monochromated MoKa radiation (λ = 0.71073 Å). Experimental data reduction was performed using APEX2 suite (Bruker AXS Inc., Billerica, MA, USA), SAINT (version 8.18c), and SADABS (version 2.11). The structure was resolved by direct methods and refined by the full-matrix least-squares method against F^2^ in the anisotropic–isotropic approximation. The H atom positions of OH groups were located from difference maps and refined isotropically. The rest of the hydrogen atoms were calculated with the riding model. All calculations were performed using SHELXTL-2018/3. CCDC 2201817 contains the supplementary crystallographic data for this paper. These data can be obtained free of charge from the Cambridge Crystallographic Data Centre via http://www.ccdc.cam.ac.uk/conts/retrieving.html (accessed on 22 September 2022), or from the CCDC, 12 Union Road, Cambridge CB2 1EZ, UK; Fax: +44-1223-336033; E-mail: deposit@ccdc.cam.ac.uk).

Crystal data for **1**: C_8_H_12_N_8_O_4_, M = 284.26, orthorhombic, space group *Pbca*, at 296 K: *a* = 14.2788(10), *b* = 9.4362(5), *c* = 17.5895(12) Å, *V* = 2370.0(3) Å^3^, *Z* = 8, *d*_calc_ = 1.593 g·cm^−3^, μ = 0.130 mm^−1^, a total of 32024 measures (*θ*_max_ = 26.036◦), 2335 unique (*R*_int_ = 0.0797), 1698 [*I* > 2σ(*I*)], 189 parameters. GooF = 1.028, *R*_1_ = 0.0358, *wR*_2_ = 0.0825 [*I* > 2σ(*I*)], *R*_1_ = 0.0610, *wR*_2_ = 0.0958 (all data), max/min diff. peak 0.19/−0.23 e·Å^−3^.

**Figure 3:**
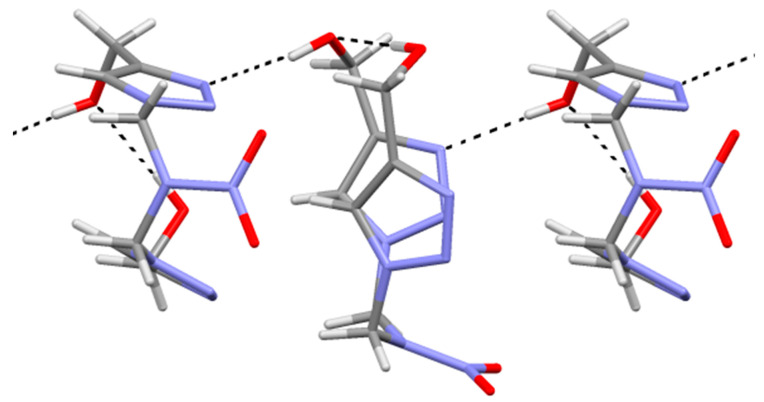
Hydrogen bonding in the crystal of compound **1**.

Molecular chains are formed in the crystal along the b-axis using the hydrogen bonds O1–H…N3 (H…O 1.96(2), O…N 2.807(2) A,O–H…N 177(2)°) (Figure 3).

### 2.6. Antiarrhythmic Activity Testing of Compound ***1***

The effect of 2-nitro-1,3-bis(4,4′-dihydroxymethyl)-1,2,3-triazolyl-2-azapropane (**1**) on the change in ECG parameters was studied, and the compound was shown to have no statistically significant impact on the basic ECG parameters in rats (Table 9).

**Figure 4:**
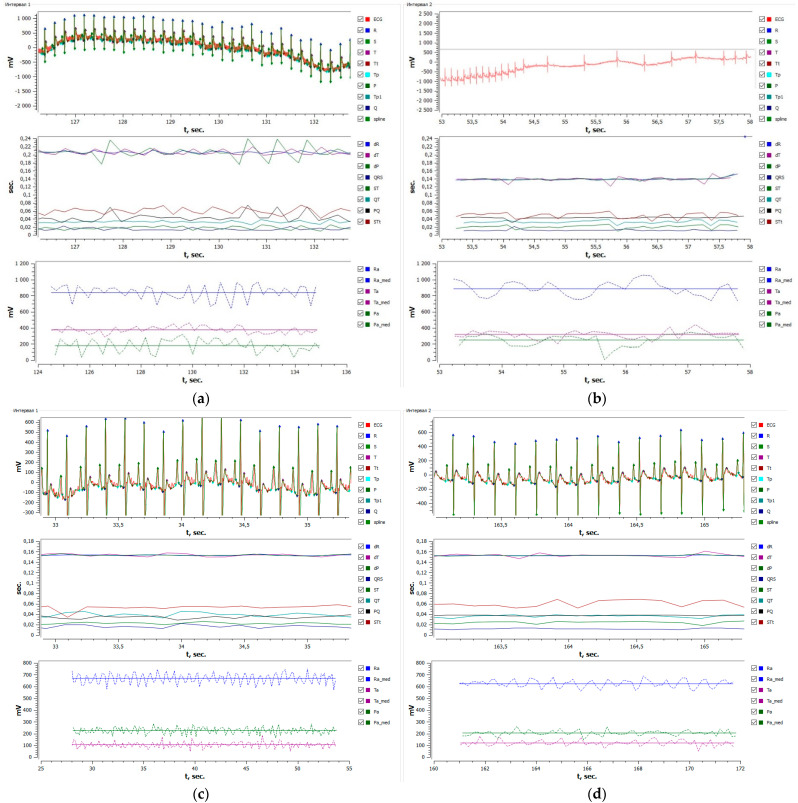
Restoration of ECG in the rat after CaCl_2_ injection on the background of compound 1 at a 0.25 mg/kg dose: (**a**) native ECG, (**b**) arrhythmia secondary to CaCl_2_ injection, (**c**) ECG after injection of compound **1**, and (**d**) restoration of ECG in the rat when CaCl_2_ was injected on the background of compound **1** at a 0.25 mg/kg dose (ED_50_).

Compound **1** was tested for antiarrhythmic activity at doses of 5.0, 0.5, 0.25, and 0.05 mg/kg against arrhythmia models provoked by calcium chloride and epinephrine (Table 10, Figure 4).

It is seen from Table 10 that compound **1** presents a pronounced antiarrhythmic activity towards calcium-chloride-induced arrhythmia. For instance, compound **1** injected at doses of 5.0 and 0.5 mg/kg against calcium chloride led to a 100% restoration of ECG in rats, while the percentage of survived animals at a dose of 0.25 mg/kg was 50%, i.e., the median effective dose (ED_50_). This agent at a dose of 0.05 mg/kg exhibited no antiarrhythmic activity. The restored ECG secondary to compound **1** at a dose of 0.25 mg/kg after a calcium chloride injection is depicted in Figure 4.

Compound **1** at a dose of 5.0 mg/kg tested towards epinephrine-induced arrhythmia resulted in a 100% lethality of animals in all the groups, suggestive of no effect in this model.

For acute toxicity it was demonstrated that no intoxication pattern was observed in animals for 14 days at a 1000 mg/kg dose, and there was no lethality (Table 11).

Thus, the tests of compound **1** demonstrated its dose-dependent effect against calcium-chloride-induced arrhythmia but no effect against epinephrine-induced arrhythmia. The administration of compound **1** at doses of 5.0 and 0.5 mg/kg against calcium chloride resulted in a 100% restoration of ECG in the rat, while the percentage of survived animals at a dose of 0.25 mg/kg was 50%, i.e., the median effective dose (ED_50_). This activity can be due to the antiarrhythmic agent influencing the calcium channels. The compound at a 0.05 mg/kg dose had no antiarrhythmic activity towards calcium chloride, as well as towards epinephrine-induced arrhythmia. The acute toxicity test showed no toxic effect at doses from 500 to 1000 mg/kg, and allows this compound to be referred to Class III: moderately hazardous substances compliant with GOST R 12.1.007-76.

**Figure 5:**
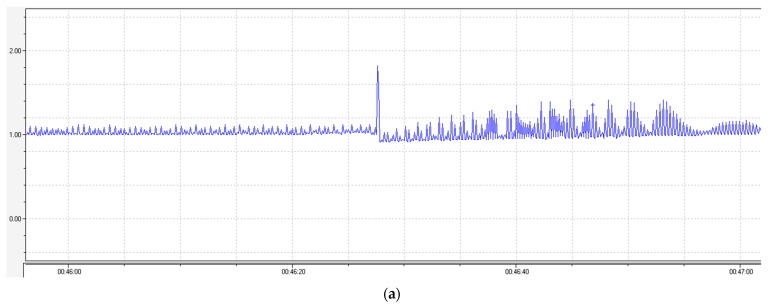

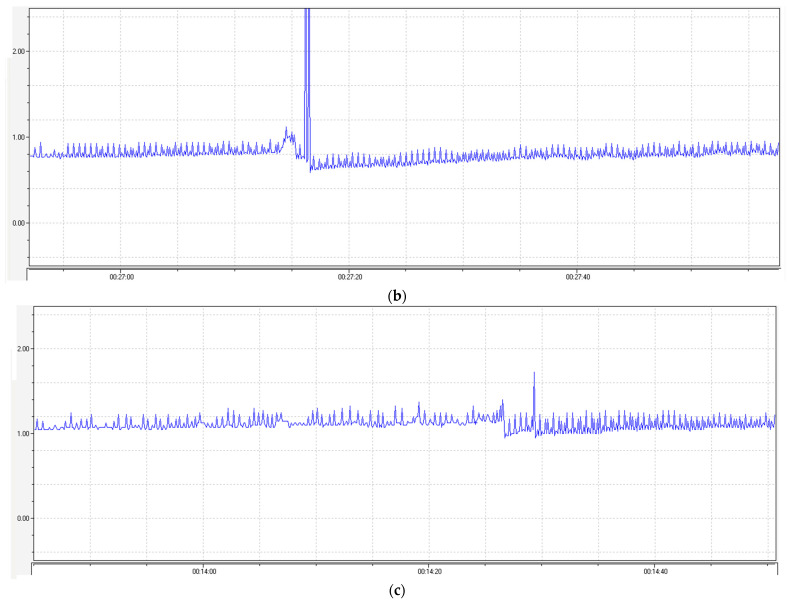
Changes in the atrial contractions of the rat heart when CaCl_2_ was injected at a dose of (**a**) 10^−3^ M, (**b**) 10^−3^ M on the background of agent **1** at a concentration of 10^−4^ M, and (**c**) 10^−3^ M on the background of nifedipine at a concentration of 10^−4^ M. The peak shows the injection site of CaCl_2_.

The findings demonstrated that compound **1** can block arrhythmia induced by an injected CaCl_2_ lethal dose. The extirpated auricle of the rat heart was employed to investigate the action mechanism. Nifedipine, a well-known antiarrhythmic drug blocking the calcium channels, was used as the reference. Figure 5a–c display changes in the auricle contractions when (a) CaCl_2_ was injected at a dose of 10^−3^ M, (b) CaCl_2_ was injected at a dose of 10^−3^ Mon the background of agent **1** at a concentration of 10^−4^ M, and (c) CaCl_2_ was injected at a dose of 10^−3^ Mon the background of nifedipine at a concentration of 10^−4^ M.

No significant changes in the contraction were observed when the test agent **1** was injected into a cuvette with the auricle of heart, but when CaCl_2_ was injected, no changes in the atrial contractions typical of the CaCl_2_ injection were observed on the background of agent **1** (Figure 5b). The CaCl_2_ injection on the background of nifedipine evoked a significant increase in the contraction amplitude of the rat auricle, as in the case of the agent **1** injection (Figure 5c). The data on the changes in the contraction amplitude and frequency are listed in Table 12.

It is seen from Table 12 that the contraction frequency was increased negligibly by the nifedipine injection, as opposed to agent **1**. The obtained data suggest that agent **1** is a calcium-channel blocker, which is in full agreement with in vivo data.

## 3. Materials and Methods

### 3.1. General Information

Infrared spectra of the samples were recorded in KBr on an FT-801 Fourier spectrometer (Simex, Novosibirsk, Russia) at 4000 to 500 cm^−1^. Elemental analysis was performed on a Flash EA 1112 series CHNS-O analyzer (ThermoQuest Italia S.p.A., Milan, Italy). Melting temperatures were measured on a Stuart SMP 30 melting point apparatus (Bibby Scientific Ltd., Stone, UK). The decomposition temperature was measured on TGA/SDTA 851e and DSC 822e thermal analyzers (Mettler Toledo, Zurich, Switzerland) over a temperature range of 25–600 °C under nitrogen at a heating rate of 10 °C/min. The results were digitized and processed in STARe 11.0 thermal analysis software. ^1^H (400 MHz) and ^13^C (100 MHz) NMR spectra were recorded on a Bruker AM-400 spectrometer (Bruker Corporation, Billerica, MA, USA) in DMSO-d_6_ (δ_H_ 2.50 ppm, δ_C_ 39.50 ppm). The reaction was monitored by thin-layer chromatography (TLC) using Merk 60 F_254_ aluminum plates (Macherey-Nagel GmbH & Co., KG, Düren, Germany). An Agilent 1200 chromatograph (Agilent Technologies, Santa Clara, CA, USA) equipped with a degasser, a gradient pump, and a diode-array detector was used for HPLC analysis. The separation was performed on a Hypersil ODS column (2.1 × 100 mm with a 5 µm particle size). The toxicity and biological activity of compound **1** were tested at the Laboratory for Pharmacological Studies in the NIOCh SB RAS.

The starting 1,3-diazido-2-nitro-2-azapropane was synthesized by the procedure reported previously [16]. The synthesis of 1,5-diazido-3-nitrazapentane was effected by the method reported in [26,27]. Copper wire of 0.4 mm in diameter (Table 1, entry 1) was cut into pieces 1–2 mm long. Copper powder (Table 1, entry 2) was prepared as described in [23]. Copper powder (Table 1, entry 3) was acquired from AO Uralelectromed (Verkhnyaya Pyshma city, Russia). Nanoscale copper oxide of 2–10 nm in size (Table 1, entry 4) was prepared as reported in [24,25]. Nanoscale copper oxide of 50–100 nm in size (Table 1, entry 5) was acquired from Advanced Powder Technologies LLC (Tomsk, Russia). Powdered copper oxide (Table 1, entry 6) was the reagent compliant with GOST R No. 16539-79. Propargyl alcohol and phenylacetylene were procured from Thermo Fisher Scientific.

HPLC chromatographic conditions were as follows. Mobile phase (*v*/*v*): A: 0.2% phosphoric acid, B: acetonitrile; gradient mode: 2% B for 5 min, 2% to 40% B in 10 min, 40% to 70% B in 5 min, and 70% B for 5 min; running time 25 min; flow rate 0.3 mL/min; column temperature 25 °C; detection UV 235 nm; injection volume 5 µL; sample concentration 1 mg/mL.

### 3.2. Synthesis

#### 3.2.1. General Synthetic Protocol for 1,4-Substituted 1,2,3-Triazoles (**1**–**4**)

Alkyne (0.0066 mol) and copper powder (0.006 mol) were weighed successively into a 12% solution of azidoalkyl nitramine (0.003 mol) in methylene chloride with stirring and held at room temperature until the starting azide completely disappeared from the reaction mixture (TLC monitoring, 3.5:1.5 benzene:methanol as the eluent). Acetonitrile was then poured into the reaction mixture and heated until the sediment was completely dissolved; the copper catalyst was separated by hot filtration, the stock solution was cooled down, and the precipitate was collected by filtration and air-dried. The filtrate was evaporated to dryness, and the overall yield was quantified. If necessary, the resultant compounds can be recrystallized. 

#### 3.2.2. 2.-Nitro-1,3-Bis(4,4′-Dihydroxymethyl)-1,2,3-Triazolyl-2-Azapropane (**1**) 

Yield: 69.8%. Mixed acetone/water (10:0.5) were used as the solvent. Purity: 99.9% (after two recrystallizations). Mp (capillary): 126–131 °C. IR spectrum, cm^−1^: 3380, 3197, 3154, 3105, 3054, 3026, 3003, 2951, 2926, 2877, 15 74, 1550, 1454, 1419, 1361, 1292, 1273, 1221, 1177, 1155, 1125, 1093, 1064, 1050, 1035, 1007, 944, 932, 879, 836, 783, 750, 710, 624. Mp: 114.66 °C (DSC). ^1^H NMR (400 MHz, DMSO-d6) δ 8.13 (s, 2H), 6.55 (s, 4H), 5.26 (t, *J* = 6.0 Hz, 2H), 4.53 (d, *J* = 8.0 Hz, 4H). ^13^C NMR (100 MHz, DMSO-d6): δ 148.63, 123.96, 62.86, 55.28. Calcd. for C_8_H_12_N_8_O_4_: C, 33.8%; H, 4.22%; N, 39.43%; found: C, 34.02%; H, 4.28%; N, 39.12%.

#### 3.2.3. 3.-Nitro-1,5-Bis(4,4′-Dihydroxymethyl)-1,2,3-Triazole-3-Azapentane (**2**)

Yield: 94.1%. Mixed acetone/water (10:1.9) was used as the solvent. Purity: 99.0% after recrystallization. Mp (capillary): 157–159 °C. IR spectrum, cm^−1^: 3300, 3160, 2961, 2859, 2756, 1568, 1494, 1457, 1434, 1420, 1372, 1339, 1326, 1277, 1238, 1179, 1142, 1066, 1046, 1022, 974, 938, 925, 829, 793, 769, 750, 680, 627. ^1^H NMR (400 MHz, DMSO-d6) δ 8.00 (s, 2H), 5.22 (t, *J* = 6.0 Hz, 2H), 4.56 (t, *J* = 6.0 Hz, 4H), 4.51 (d, *J* = 8.0 Hz, 4H), 3.97 (t, *J* = 6.0 Hz, 4H). ^13^C NMR (100 MHz, DMSO-d6): δ 148.64, 123.81, 55.35, 51.79, 46.31. The IR and NMR spectroscopic data are in agreement with the literature [1].

#### 3.2.4. 2.-Nitro-1,3-Bis(4,4′-Diphenyl)-1,2,3-Triazolyl-2-Azapropane (**3**)

Yield: 79.0%. Purity: 99.95%. Mp (capillary): 251–254 °C. IR spectrum, cm^−1^: 3131, 3105, 3049, 3017, 2951, 1578, 1484, 1461, 1424, 1402, 1355, 1282, 1229, 1175, 1098, 1080, 1037, 975, 938, 862, 825, 808, 761, 694, 677, 657, 617. ^1^H NMR (400 MHz, DMSO-d6) δ 8.75 (s, 2H), 7.87 (d, *J* = 8.0 Hz, 4H), 7.34-7.48 (m, 6H), 6.66 (s, 4H). ^13^C NMR (100 MHz, DMSO-d6): δ 146.92, 130.68, 129.42, 128.62, 125.77, 122.59, 62.94. Calcd. for C_18_H_16_N_8_O_2_: C, 57.44%; H, 4.25%; N, 29.78%; found: C, 56.87%; H, 4.23%; N, 29.05%.

#### 3.2.5. 3.-Nitro-1,5-Bis(4,4′-Diphenyl)-1,2,3-Triazolyl-3-Azapropane (**4**)

Yield: 62.2%. Purity: 99.78%. Mp (capillary): 239–242 °C. IR spectrum, cm^−1^: 3130, 3098, 3054, 3032, 2955, 1609, 1502, 1468, 1444, 1417, 1381, 1338, 1274, 1226, 1203, 1080, 1026, 1004, 974, 917, 812, 765, 692. ^1^H NMR (400 MHz, DMSO-d6) δ 8.59 (s, 2H), 7.83 (d, *J* = 8.0 Hz, 4H), 7.32-7.47 (m, 6H), 4.67 (t, *J* = 6.0 Hz, 4H), 4.10 (t, *J* = 6.0 Hz, 4H). ^13^C (100 MHz, DMSO-d6): δ 146.97, 131.02, 129.39, 128.42, 125.62, 122.46, 51.85, 46.78.: Calcd. for C_20_H_20_N_8_O_2_: C, 59.40%; H, 4.95%; N, 27.72%; found: C, 58.69%; H, 4.87%; N, 26.86%.

### 3.3. Materials and Methods for Bioassay 

#### 3.3.1. Toxicology

Acute toxicity was measured on 20 outbred CD-1 mice (10 mice per a group) weighing 25–30 g by *per os* administration at doses from 500 to 1000 mg/kg.

Statistical analysis was performed using STATISTICA 12.0 software (Informer Technologies Inc., Los Angeles, CA, USA) and a software designed by the NIOCh SB RAS to estimate ECG parameters.

#### 3.3.2. Antiarrhythmic Activity

The antiarrhythmic activity was tested on adult male Wistar rats. All the animals were acquired from the SPF vivarium of the Institute of Cytology and Genetics of the Siberian Branch of Russian Academy of Sciences (IC&G SB RAS), where they were housed under standard conditions with ad libitum access to water and standard rodent pellet diet. After quarantine, the animals were randomized by weight and split into groups of 10 species each.

Experiments with animals were performed in strict adherence to Order No. 199n “Guidelines for Good Laboratory Practice” issued by the Ministry of Health of the Russian Federation on 1 April 2016, and to the EU Directive 2010/63/EU “On the Protection of Animals Used for Scientific Purposes”. All the experimental procedures were approved by the Bio-Ethical Committee of the Novosibirsk Institute of Organic Chemistry of the Siberian Branch of Russian Academy of Sciences in accordance with the 2010 European Convention for the Protection of Vertebrate Animals used for Experimental and other Scientific Purposes, and with the Guide for the Care and Use of Laboratory Animals. 

The rats were narcotized by intraperitoneal injection of thiopental sodium (0.12 mg/kg) and then fixated to record electrocardiogram (ECG). For this, three electrodes were attached to the rat body to record the second standard limb lead. The electrodes were connected to an ECG V75-11 isolated amplifier (Coulbourn Instruments, Whitehall, PA, USA). The recording and monitoring of the RR, PQ, QRS, QT, and P time intervals were evaluated along with amplitudes of the P, T, and R waves for 10 min by using LabView 6.1 software. 

Arrhythmia was provoked either by a single 250 mg/kg dose of 10% aqueous CaCl_2_ injected intravenously or by a 0.3 mg/kg epinephrine injection to rats. These arrhythmogenic doses are lethal to rats (100% lethal dose, LD_100_). The test agents were diluted with saline and injected into the femoral vein (the same site in rats) at 5, 0.5, 0.25, and 0.05 mg/kg doses. Such an injection route allows the direct blockage of acute arrhythmia. 

#### 3.3.3. Ex Vivo Experimentation

The examination was performed on Wistar rats of both sexes weighing 200–230 g. After decapitation, the abdomen was opened to subsequently remove the heart. The right atrium was cut off using microscissors and placed into Ringer–Locke’s solution (118 mmol/L NaCl, 5.6 mmol/L KCl, 0.25 mmol/L CaCl_2_, 25 mmol/L NaHCO_3_, and 1 mmol/L glucose), preheated to 37 °C. The bath containing the removed atrium was brought to an organ bath (PanLab, Spain) and attached to a transducer on one end and to a metal rod on the other end; afterwards, it was immersed into a 10 mL cuvette filled with 95% oxygenated Ringer–Locke’s solution at 37 °C. The data analysis and recording were performed in Protowin software (PanLab Technology, Barcelona, Spain) for biological studies. The test and reference compounds were dissolved in physiological saline at concentrations of 1 × 10^−4^ M.

## 4. Conclusions

A synthetic process for 2-nitro-1,3-bis(4,4′-dihydroxymethyl)-1,2,3-triazolyl-2-azapropane (**1**) was developed for the first time and relies on the 1,3-dipolar cycloaddition reaction between 1,3-diazido-2-nitro-2-azapropane (DANP) and propargyl alcohol over a copper-based catalyst (copper powder). The optimum reaction conditions were identified. Based on this approach, new 1,2,3-triazole derivatives (**3** and **4**) were synthesized. The structures of the resultant compounds were confirmed by IR and NMR spectroscopies and elemental analysis. The structure of compound **1** was proved by single-crystal X-ray diffraction. The tests demonstrated that compound **1** exhibits a dose-dependent antiarrhythmic activity towards calcium-chloride-induced arrhythmia and refers to Class III: moderately hazardous substances. 

## Figures and Tables

**Table 1:** Effect of catalysts on reaction time, yield, and quality of **1**.

Entry	Catalyst	Reaction Time, h	Yield of Crystalline 1, %	Total Yield of 1 ^1^, %	Purity, %
1	Copper wire	55.0	43.0	52.0	90.87
2	Copper powder [23]	15.0	37.0	53.3	98.78
3	Copper powder	9.5	47.8	57.5	97.97
4	n-CuO (2–10 nm) [24,25]	10.0	58.5	71.0	96.08
5	n-CuO (50–100 nm)	11.5	50.0	59.0	95.60
6	CuO	28.0	29.0	39.5	91.26

^1^ Total yield given with allowance for the product isolated from the filtrate.

**Table 2:** Temperature effect on reaction time and yield of **1** in *t*-butanol.

Reaction Temperature, °C	Time, h	Yield of Crystalline 1 ^1^, %	Total Yield of 1 ^1,2^_,_ %
25	22.0	– ^3^	63.2
35	9.5	46.8	54.6
50	2.0	60.7	65.6
70	1.5	41.8	56.6

^1^ Yields given with allowance for the major product content measured by HPLC. ^2^ Total yield of **1** estimated as the sum of quantities in the crystalline product and filtrate, as per HPLC. ^3^ No crystallization of **1** occurred.

**Table 3:** Effects of solvents on reaction time and yield of compound **1**.

Entry	Solvent	Reaction Time, h	Yield of Crystalline 1 ^1^, %	Total Yield of 1 ^1,2^, %
1	*t*-Butanol	22.0	–	63.2
2	Acetonitrile	6.0	30.6	44.5
3	Methanol	4.0	27.2	55.2
4	Ethanol	4.0	20.0	56.4
5	Acetone	11.0	46.4	65.8
6	Methylene chloride	6.0	56.8	68.1
7	Chloroform	3.0	63.5	70.1
8	Toluene	1.3	33.7	40.9
9	THF	4.0	34.1	57.1

^1^ Yields given with allowance for the major product content measured by HPLC. ^2^ Total yield of **1** estimated as the sum of quantities in the crystalline product and filtrate, as per HPLC.

**Table 4:** Yields of **1** and **2** subject to the azide:alkyne molar ratio.

Entry	Starting Azide	Reaction Product	Molar Ratio (Azide:Alkyne)	Reaction Time, h	Yield of Crystalline 1 ^1^, %	Total Yield of 1 ^1,2^, %
1	DANP	**1**	1:2.0	8.0	49.8	58.1
2			1:2.2	2.5	69.8	84.0
3			1:2.4	1.5	57.0	68.5
4	DIANP	**2**	1:2.0	24.0	85.8	87.9
5			1:2.2	24.0	94.1	95.6
6			1:2.4	24.0	91.6	93.1

^1^ Yields given with allowance for the major product content measured by HPLC. ^2^ Total yield of **1** estimated as the sum of quantities in the crystalline product and filtrate, as per HPLC.

**Table 5:** Effects of solvents on yield and quality of **1** when recrystallized.

Entry	Solvent	Ratio of Solvent/Compound 1, mL/g	Yield, %	Purity, %
1	Acetonitrile	9	38.0	98.9
2	Acetone	62	54.0	99.2
3	Ethanol	6	12.0	98.1
4	IPA	32	46.0	97.3
5	IPA/water	6/0.4	28.0	96.1
6	Acetone/water	10/0.5		
		1st recrystallization	72.0	99.2
		2nd recrystallization	71.3	99.9

**Table 6:** Cyclization results of DANP and DIANP with propargyl alcohol and phenylacetylene.

Comp.	Starting Reagents Azide/Alkyne	Reaction Time, h	Yield of Crystalline Product, %	Total Yield of Product, %	Melting Point (Capillary), °C
**1**	DANP/propargyl alcohol	2.5	69.8	84.0	126–131
**2**	DIANP/propargyl alcohol	24.0	94.1	95.6	157–159
**3**	DANP/phenylacetylene	21.0	78.9	83.5	251–254
**4**	DIANP/phenylacetylene	42.0	62.2	71.2	239–242

**Table 7:** Comparative basic infrared frequencies of compounds **1**–**4**.

Comp.	Frequency, cm^−1^							
**1**	3380 *, (1361)	3054, 3026	2951, 2926, 2877, (1454)	1574 s., 1273 s.	–	1419 m.	1064 w.	1007 s., 932 s.
**2** [1]	3300	3161	2934, 2860, (1434)	1568, 1279	–	1421	–	1022
**3**	abs.	3049, 3017	2951, 2850, (1444)	1578 s., 1282 s.	1461 m., 1037, 761 s., 694 s.	1424 m.	1098 w.	–
**4**	abs.	3098, 3054, 3032	2955, 2860, (1444)	1502 s., 1274 s.	1468 m., 1004 m., 765 s., 692 s.	1417 m.	1080 w.	–
Functional groups	* *ν* (δ) OH	*ν* C–H, 1,2,3-tria zolyl	*ν* (δ) CH_2_	*ν* N–NO_2_	*ν* C=C, phenyl	*ν* N=N, 1,2,3-tria zolyl	*ν* N–N, nitramine	C–OH (C–O)

Spectral intensities: s., strong; m., medium; w., weak; abs., absent; *ν*, stretching; δ, bending. * Intramolecular H-bonding.

**Table 8:** DSC data for decomposition temperatures of **1**.

Comp.	Stage I				Stage II			
	Onset, °C	Peak, °C	Endset, °C	Specific Heat Release, J/g	Onset,°C	Peak, °C	Endset, °C	Specific Heat Release, J/g
**1**	112.7	115.6	122.3	−31.7	224.4	249.0	271.0	230.3
	143.8	145.9	153.0	−21.7	335.4	363.6	370.5	122.5

**Table 9:** Impact of compound **1** on ECG parameters in rats.

Status	dR	dT	dP	QRS	ST	QT	Ra	Ta	Pa
Baseline	0.232 ± 0.0008	0.231 ± 0.052	0.232 ± 0.012	0.017 ± 0.0008	0.022 ± 0.0009	0.039 ± 0.0008	721.04 ± 30.0	156.62 ± 9.800	218.12 ± 15.15
Post-injection	0.24 ± 0.0012	0.24 ± 0.030	0.24 ± 0.009	0.015 ± 0.0010	0.022 ± 0.0008	0.037 ± 0.0001	726.82 ± 28.8	168.37 ± 15.128	221.86 ± 12.80

± Indicates a standard error spread of the means.

**Table 10:** Antiarrhythmic activity of compound **1**.

Comp.	Dosage, mg/kg	Survived Animal Percentage, %	
		Calcium-Chloride-Induced Arrhythmia	Epinephrine-Induced Arrhythmia
**1**	5.00	100	0
	0.50	100	0
	0.25	50	0
	0.05	0	0

**Table 11:** Compound **1** tested for acute toxicity at different doses in animal groups.

Agent	Dose, mg/kg	Percentage of Survived Animals on *per os* Administration, %
Compound **1**	500	100
	700	100
	1000	100

**Table 12:** Data on changes in contraction amplitude and frequency.

Agent/Concentration, (M)	Amplitude (mV)		Frequency (Hz) Reductions	
	Before	After	Before	After
Agent **1**, 10^−4^ M	0.12 ± 0.018	0.13 ± 0.004	2.6	2.6
Nifedipine, 10^−4^ M	0.14 ± 0.023 **	0.24 ± 0.045	2.2	3.4
Calcium chloride, 10^−3^ M	0.18 ± 0.010 **	0.34 ± 0.010 **	2.6	3.2
Agent **1**, 10^−4^ M, + CaCl_2_, 10^−3^ M	0.18 ± 0.018	0.2 ± 0.030	2.2	2.2
Nifedipine, 10^−4^ M, + CaCl_2_, 10^−3^ M	0.19 ± 0.023 *	0.22 ± 0.045	2.2	2.6 *

* *p* < 0.01. ** *p* < 0.001.

## Data Availability

The data used to support the findings of this study are included in the article and Supplementary Materials.

## Supplementary Materials

The following supporting information can be downloaded at: https://www.mdpi.com/article/10.3390/ph15121443/s1. The supporting information containing IR spectra: Figures S1–S4, ^1^H,^13^C NMR spectra: Figures S5–S13, and DSC and TGA data: Figures S13–S16 for synthesized compounds.
