# Supplementary material for: Investigation of 1,4-Substituted 1,2,3-Triazole Derivatives as Antiarrhythmics: Synthesis, Structure, and Properties
Source: Pharmaceuticals (Basel). 2022 Nov 22;15(12):1443. doi: 10.3390/ph15121443 (PMC9781658; doi:10.3390/ph15121443)
Supplement: Supplementary file 1 [file pharmaceuticals-15-01443-s001.zip › pharmaceuticals-2042532-supplementary.pdf]

Supplementary Materials

Investigation of 1,4-substituted 1,2,3-triazole derivatives as antiarrhythmics: synthesis, structure and properties

Elena O. Shestakova[1], Sergey G. Il'yasov[1,*], Irina A. Shchurova[1], Vera S. Glukhacheva[1], Dmitri S. Il'yasov [1], Egor E. Zhukov [1], Arkady O. Bryzgalov[2], Tatiana G. Tolstikova[2] and Yuri V. Gatilov[2]

[1]Institute for Problems of Chemical and Energetic Technologies, Siberian Branch of the Russian Academy of Sciences (IPCET SB RAS), Biysk 659322, Russia

[2]Novosibirsk Institute of Organic Chemistry, Siberian Branch of the Russian Academy of Sciences (NIOCh SB RAS), Novosibirsk 630090, Russia

**Corresponding author. E-mail: ilysow@ipcet.ru (S.G. Il'yasov)*

Table of Contents

Fig. S1.IR spectrum of **1**........2

Fig. S2.IR spectrum of **2**........2

Fig. S3. IR spectrum of **3**........3

Fig. S4. IR spectrum of **4**........3

Fig. S5.^{1}H NMR of **1**........4

Fig. S6.^{13}C NMR of **1**........4

Fig. S7.^{1}H NMR of **2**........5

Fig. S8.^{13}C NMR of **2**........5

Fig. S9.^{1}H NMR of **3**........6

Fig. S10.^{13}C NMR of **3**........6

Fig. S11.^{1}H NMR of **4**........7

Fig. S12.^{13}C NMR of **4**........7

Fig. S13. TGA and DSC of **1**........8

Fig. S14.TGA and DSC of **2**........8

Fig. S15.TGA of **3**........9

Fig. S16.TGA and DSC of **4**........9

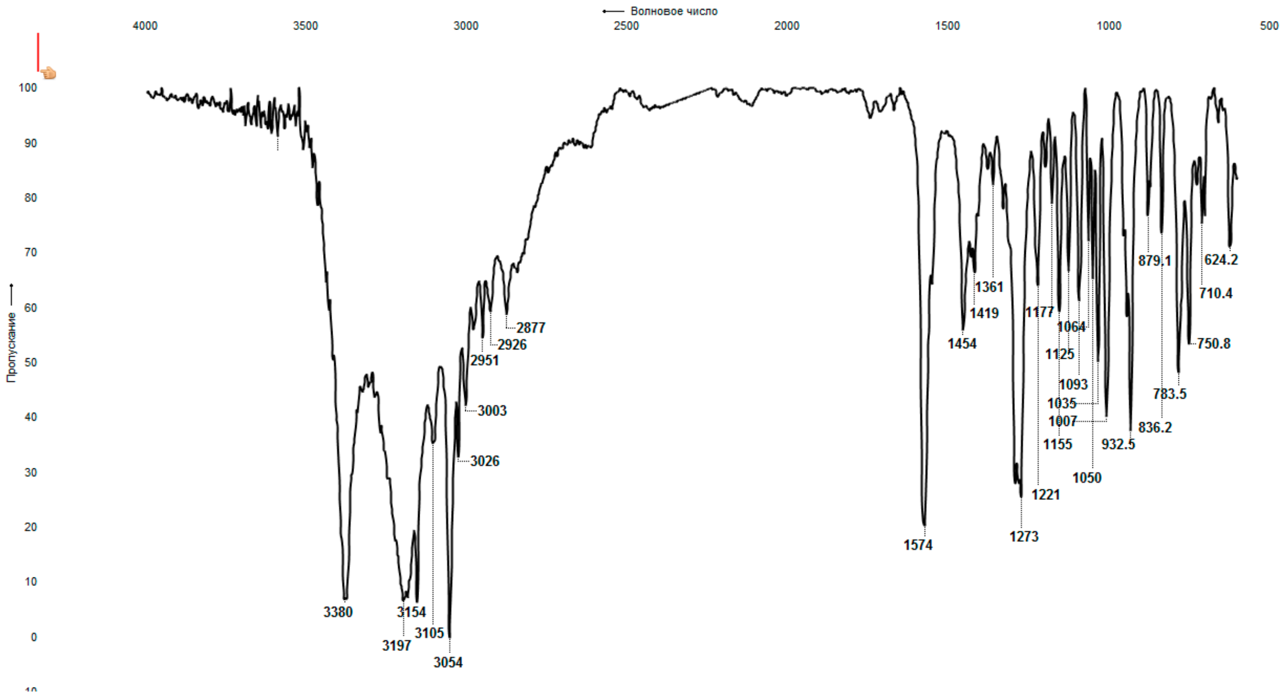

Figure S1. IR spectrum of **1**

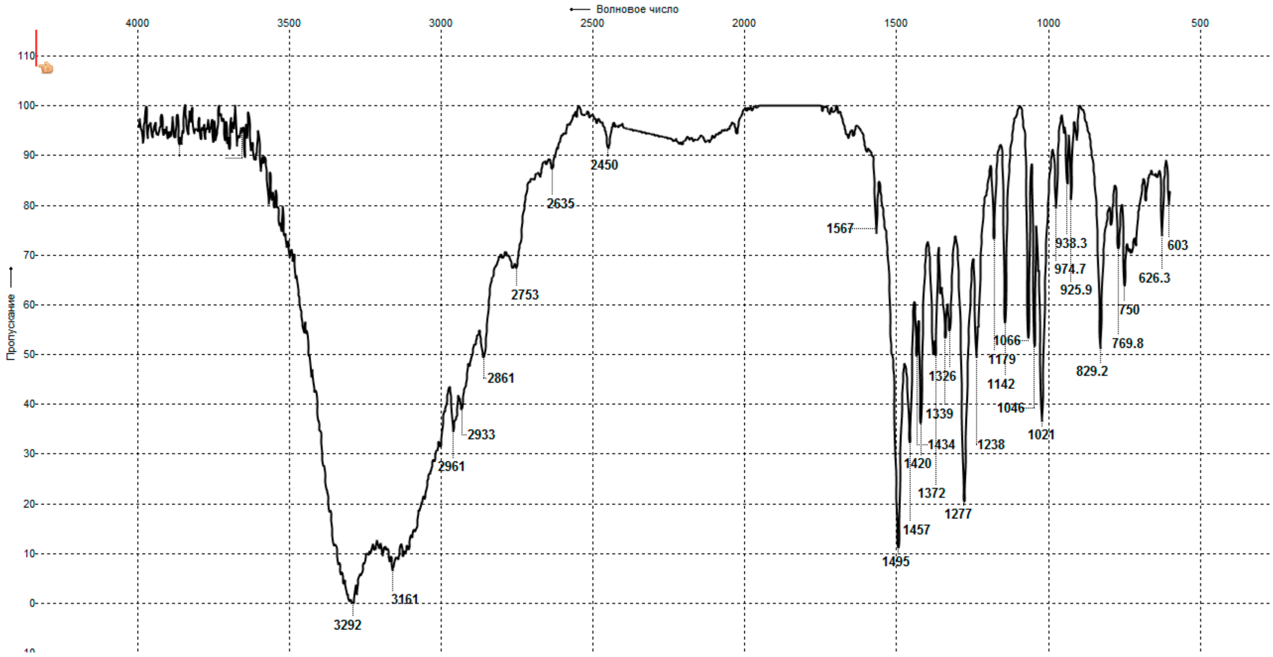

Figure S2. IR spectrum of **2**

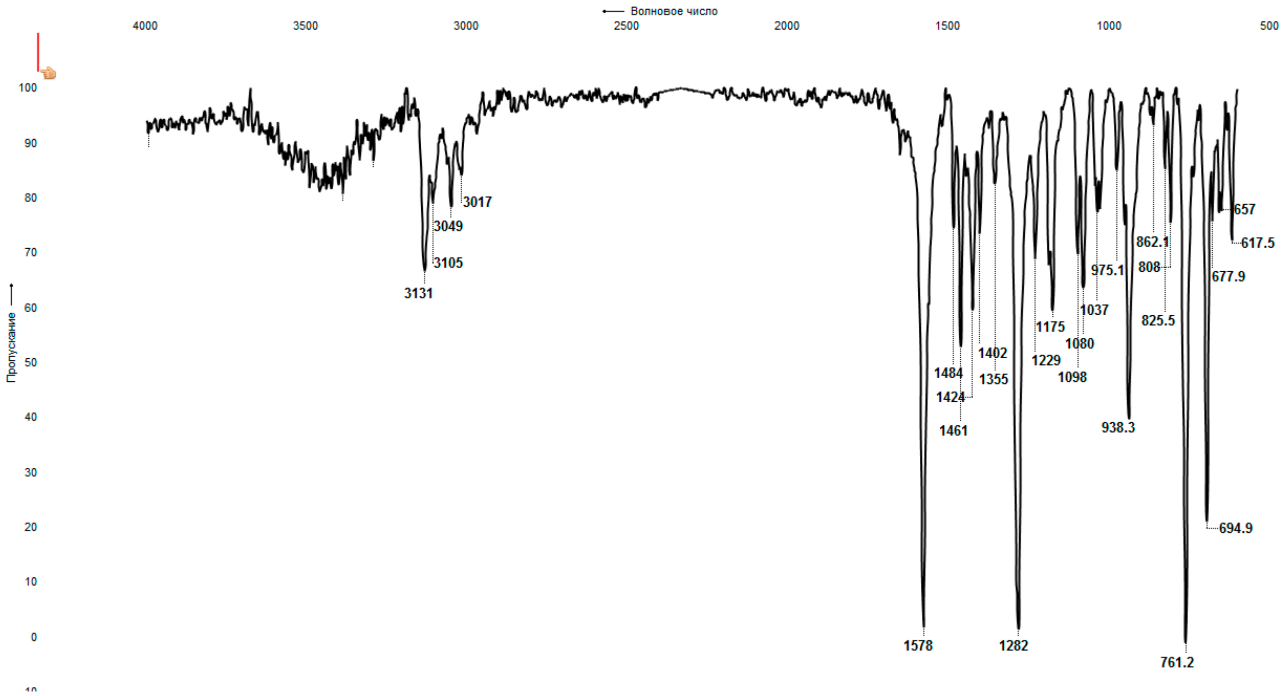

Figure S3. IR spectrum of **3**

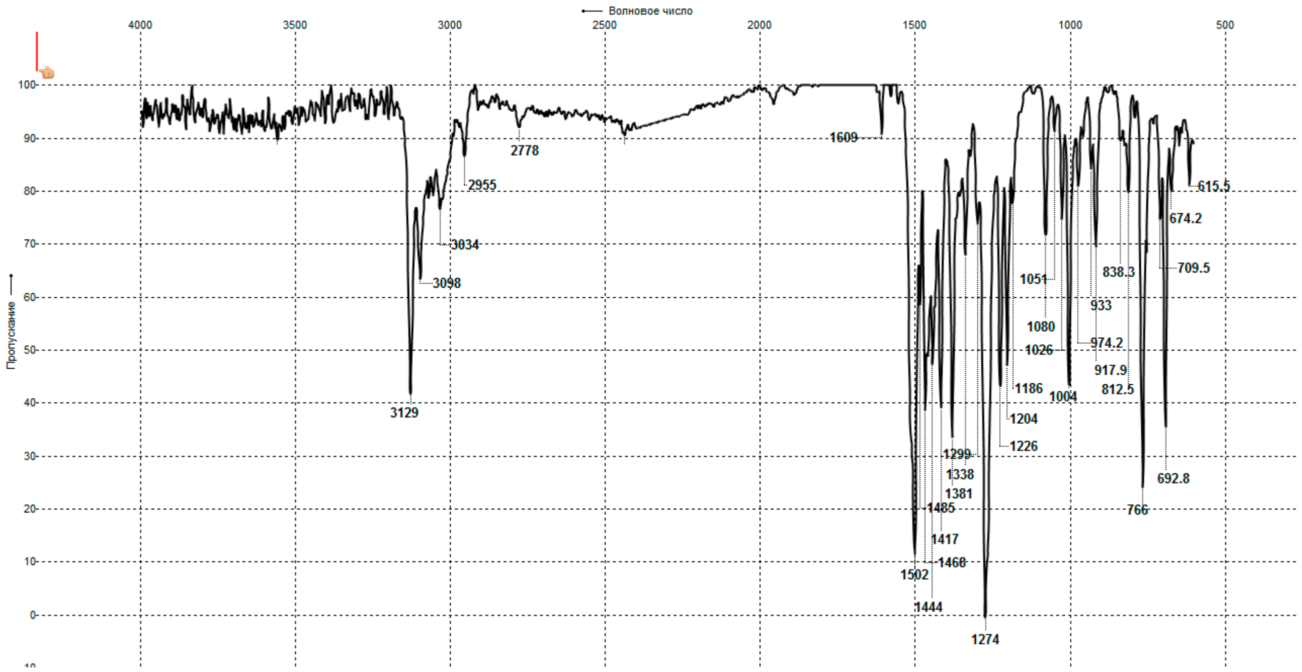

Figure S4. IR spectrum of **4**

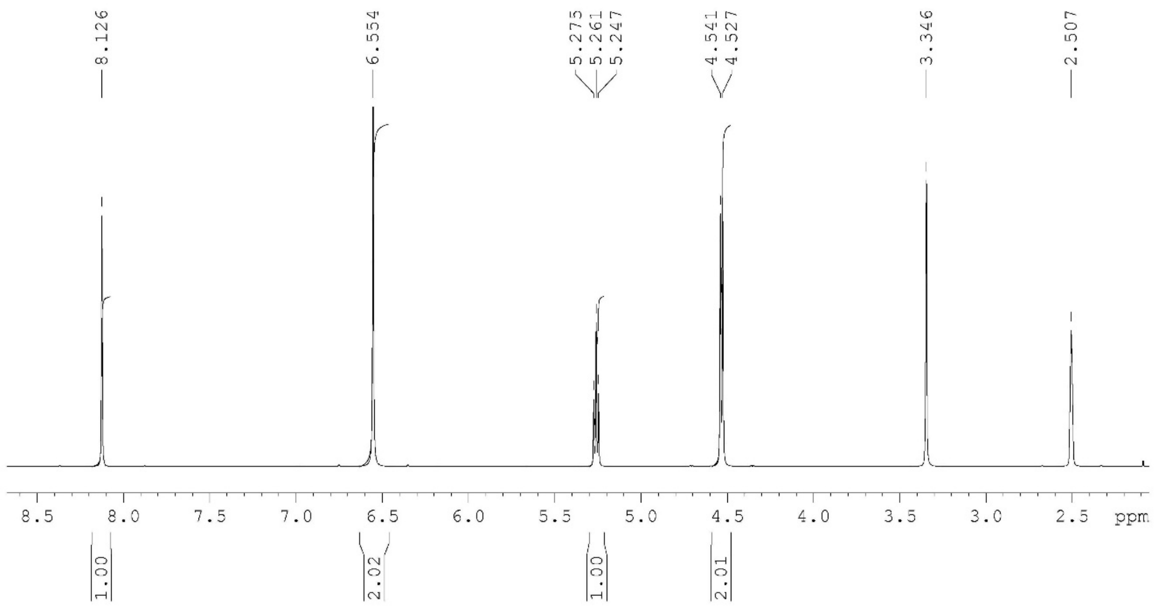

Figure S5. ^{1}H NMR of **1**

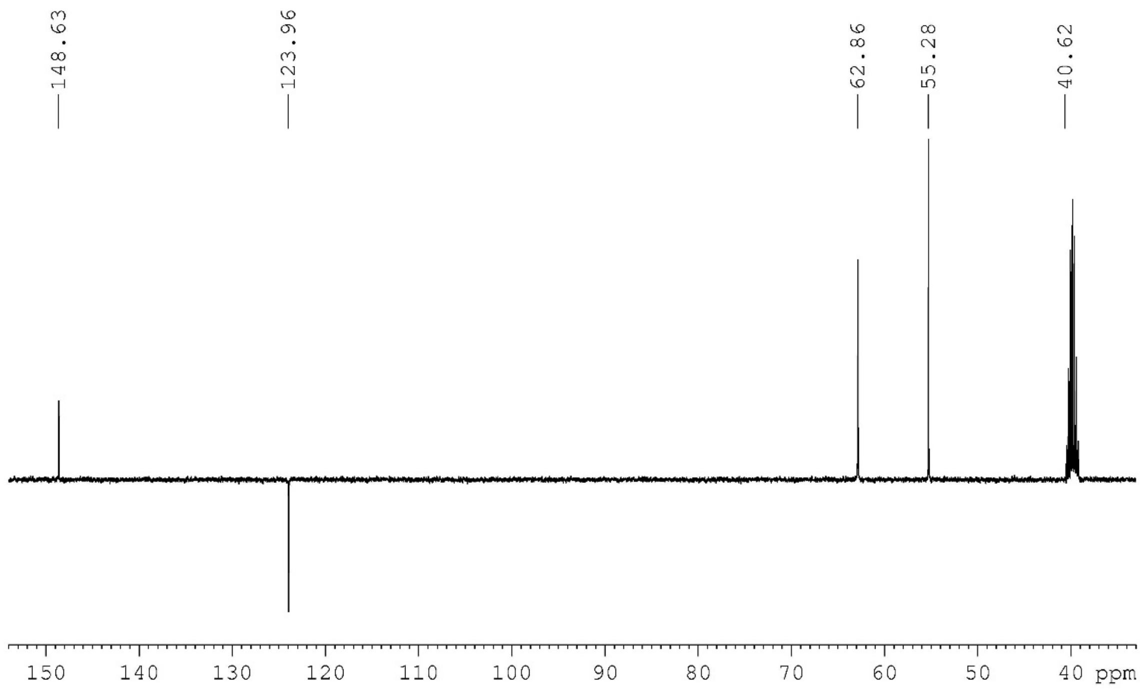

Figure S6. ^{13}C NMR of **1**

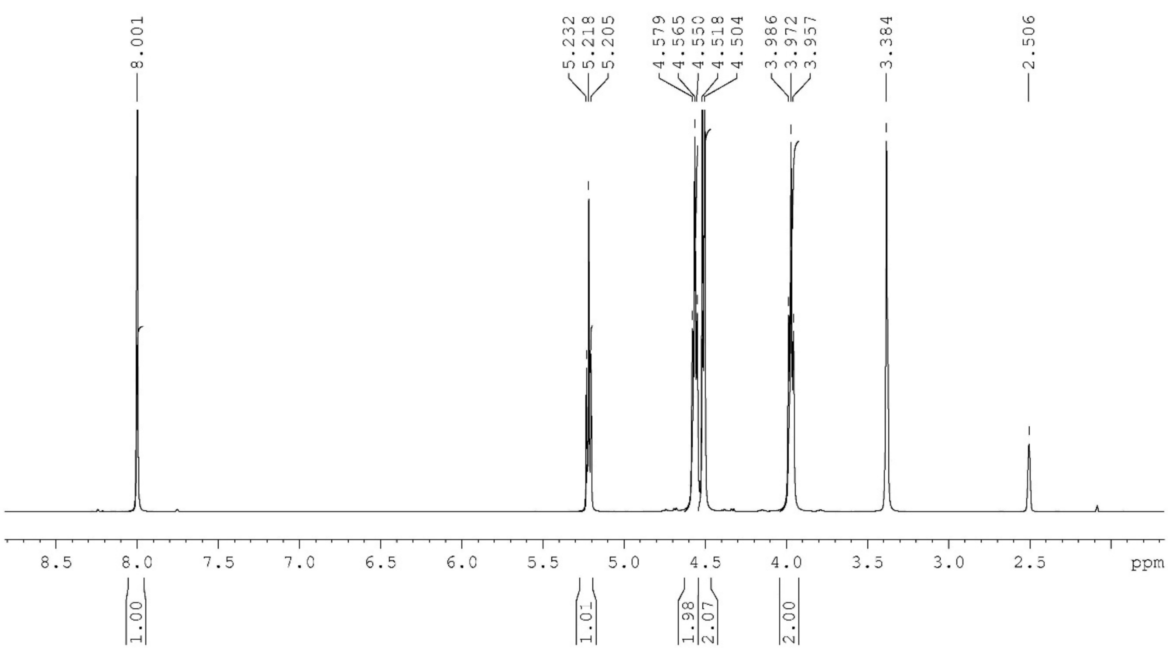

Figure S7. ^{1}H NMR of **2**

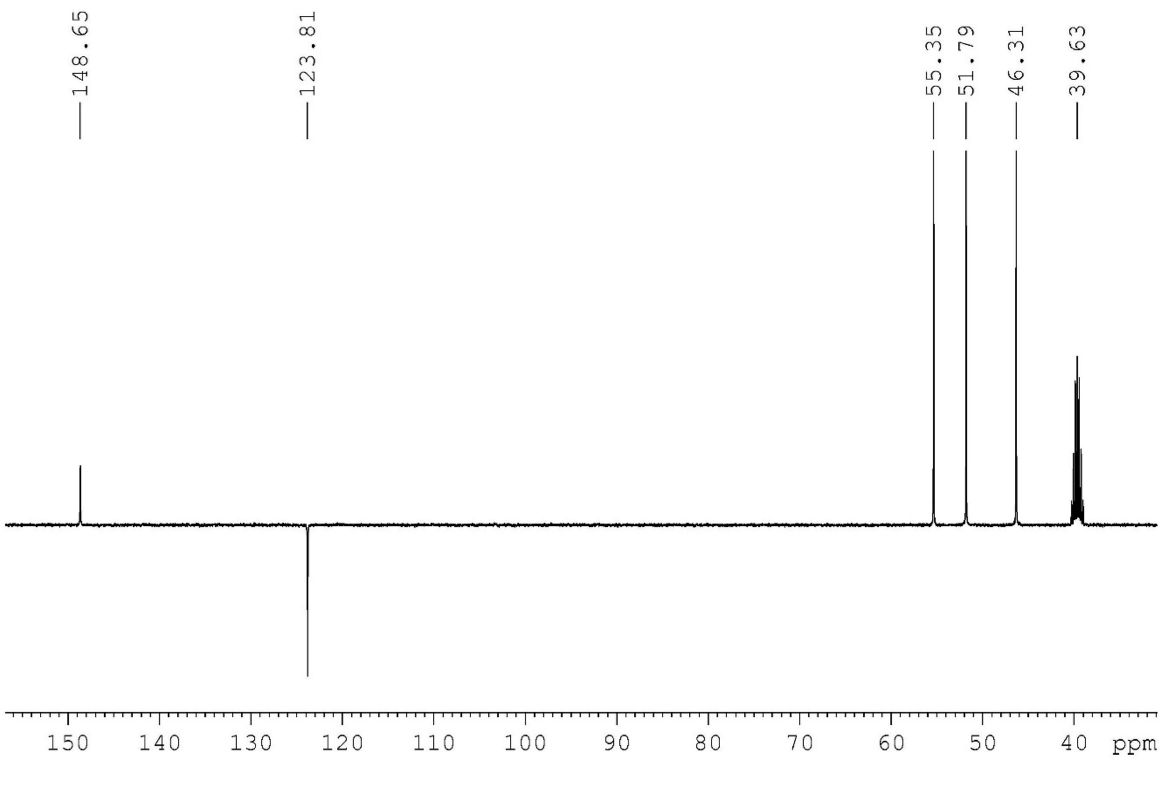

Figure S8. ^{13}C NMR of **2**

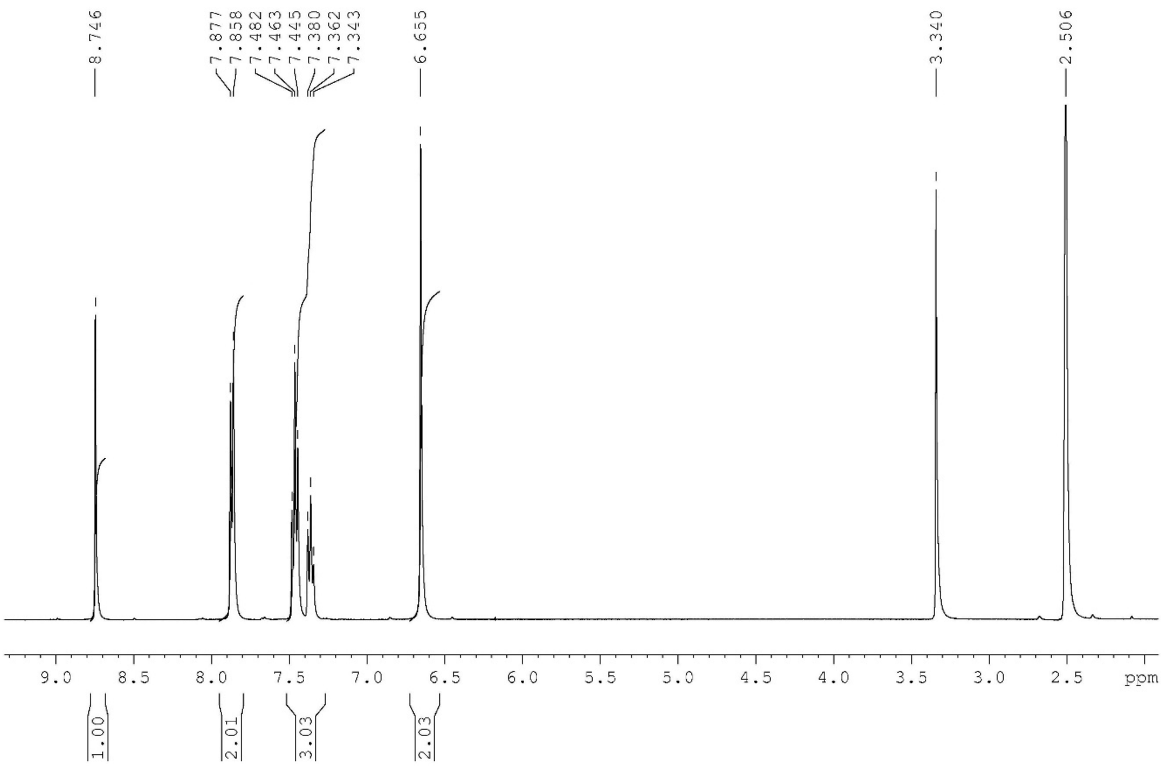

Figure S9. ^{1}H NMR of **3**

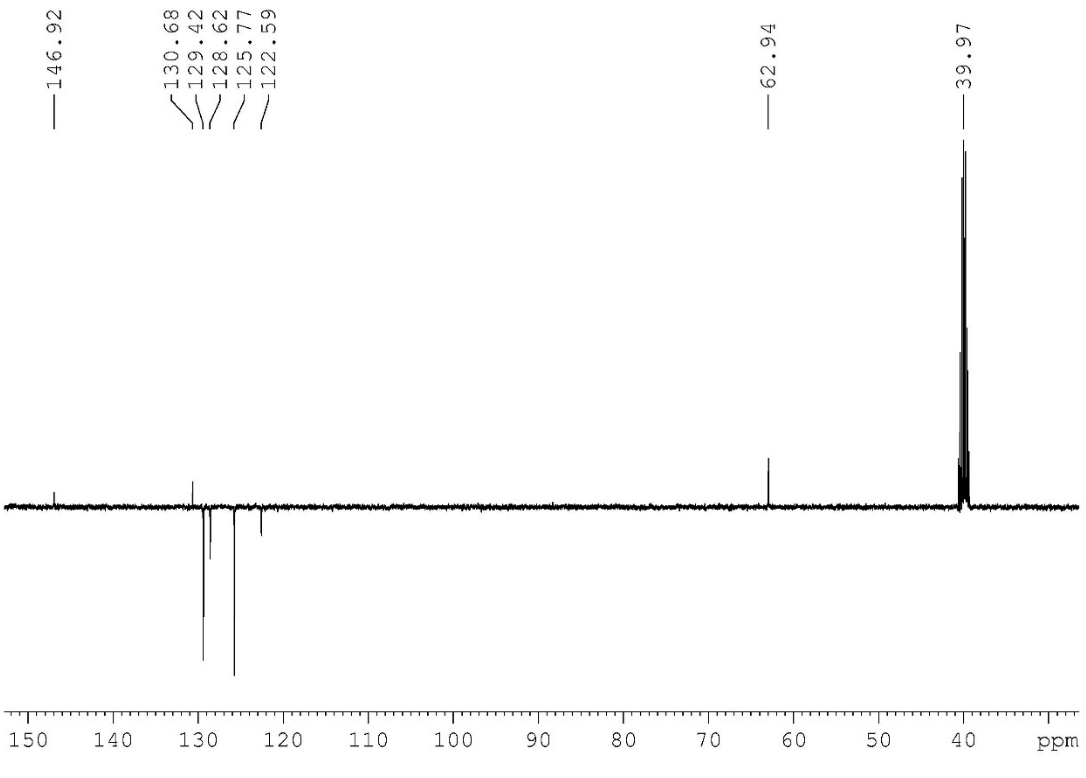

Figure S10. ^{13}C NMR of **3**

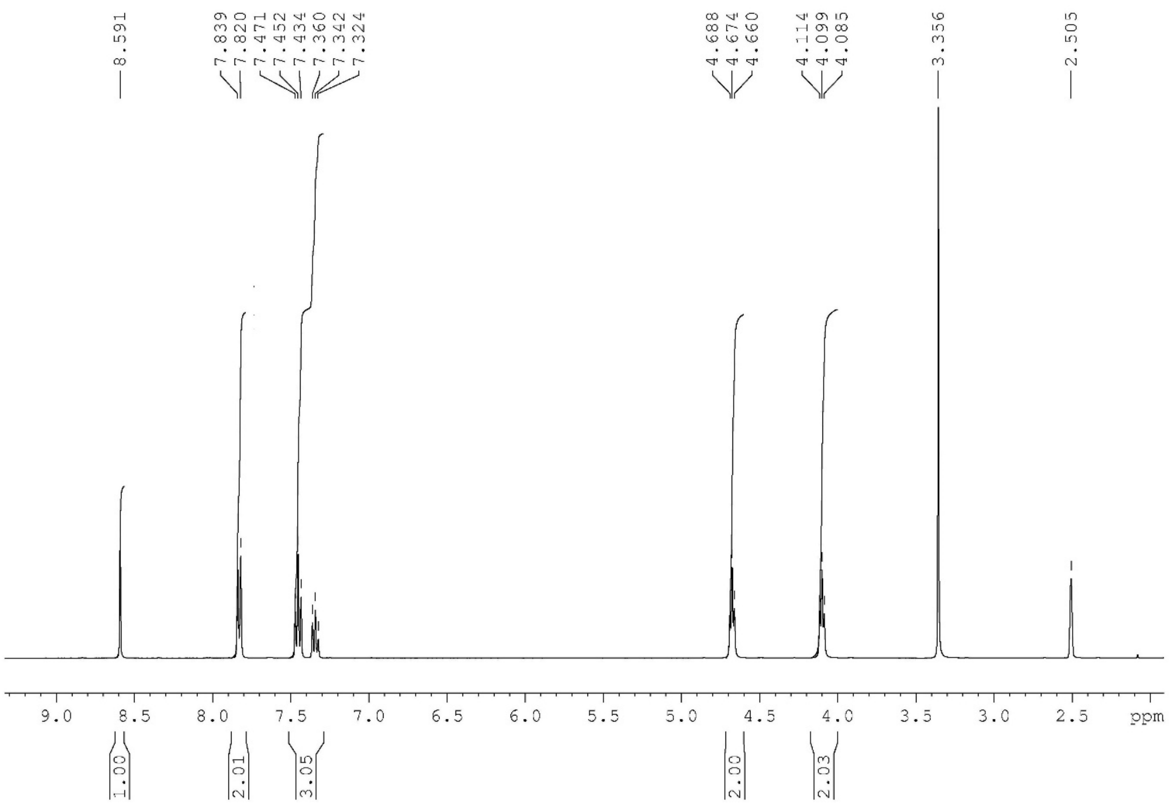

Figure S11. ^{1}H NMR of **4**

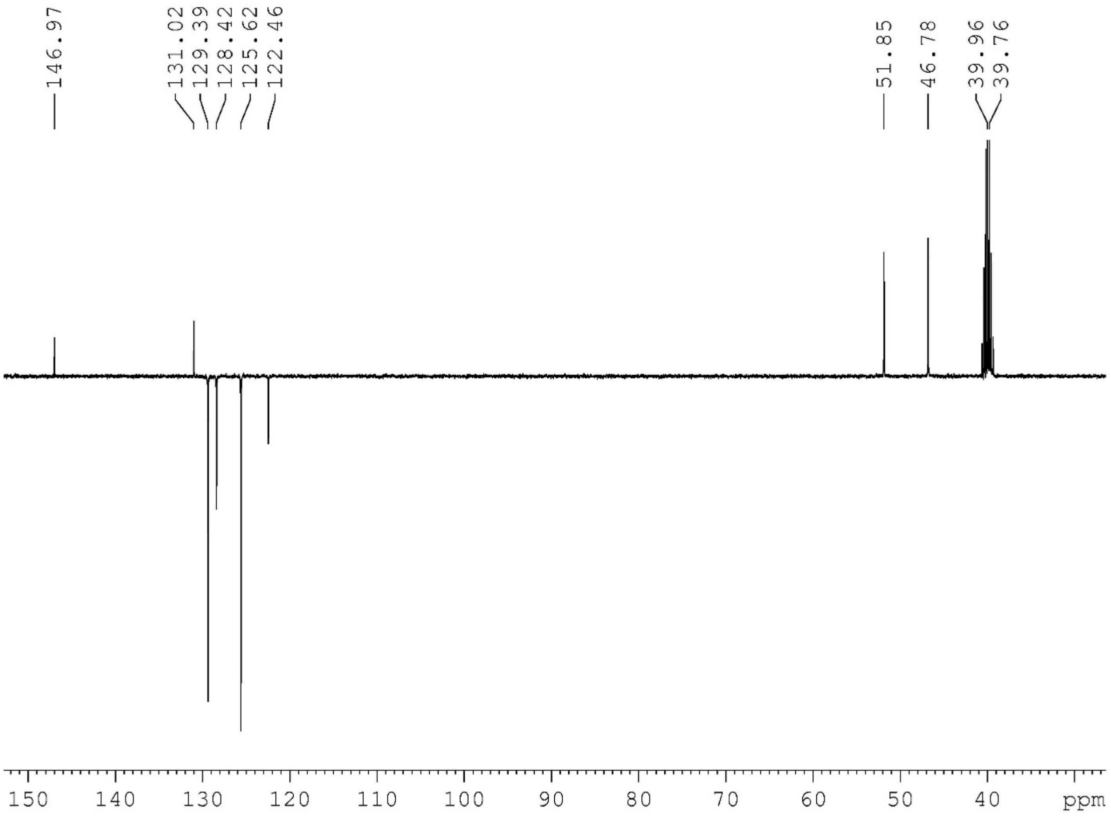

Figure S12. ^{13}C NMR of **4**

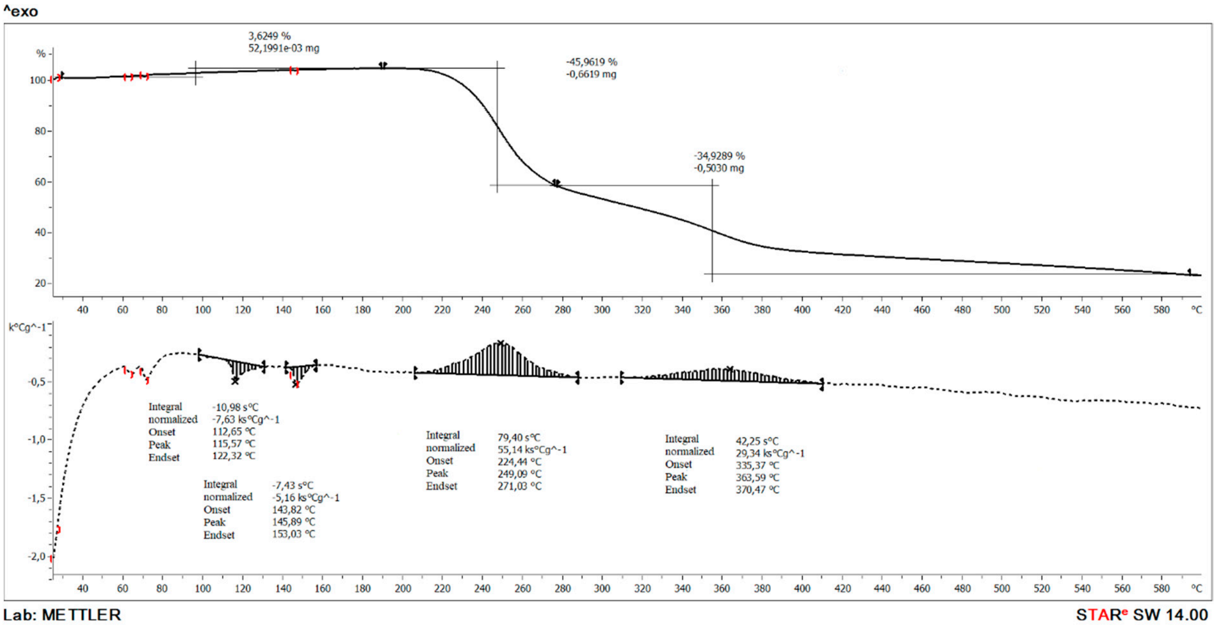

Figure S13. TGA and DSC of **1**

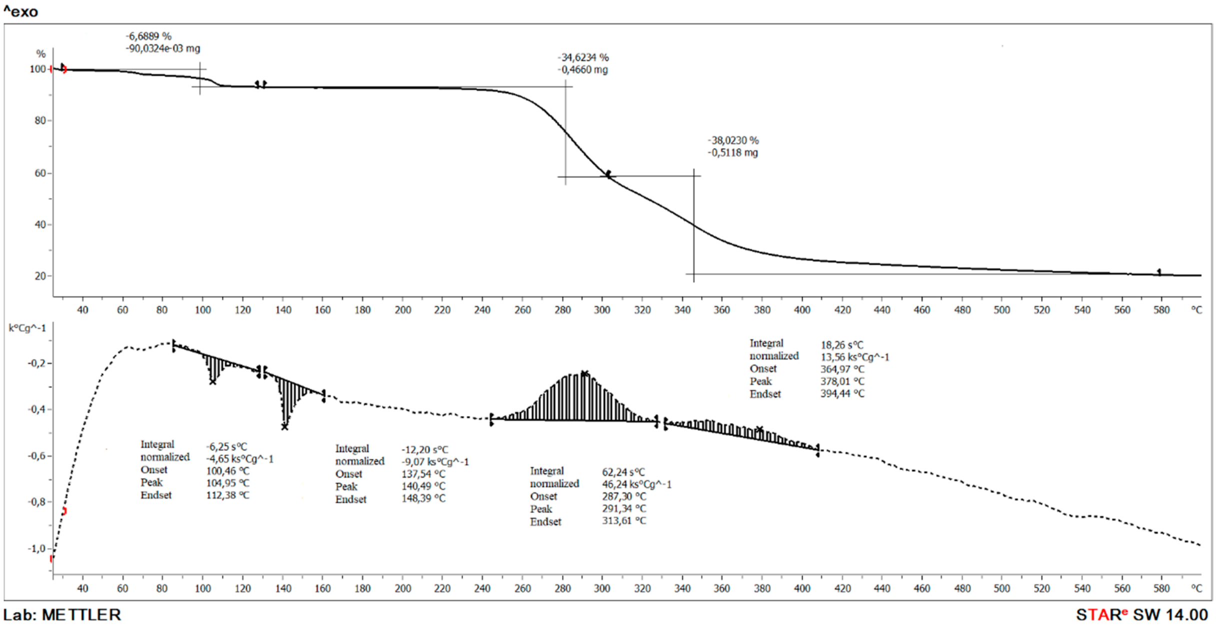

Figure S14. TGA and DSC of **2**

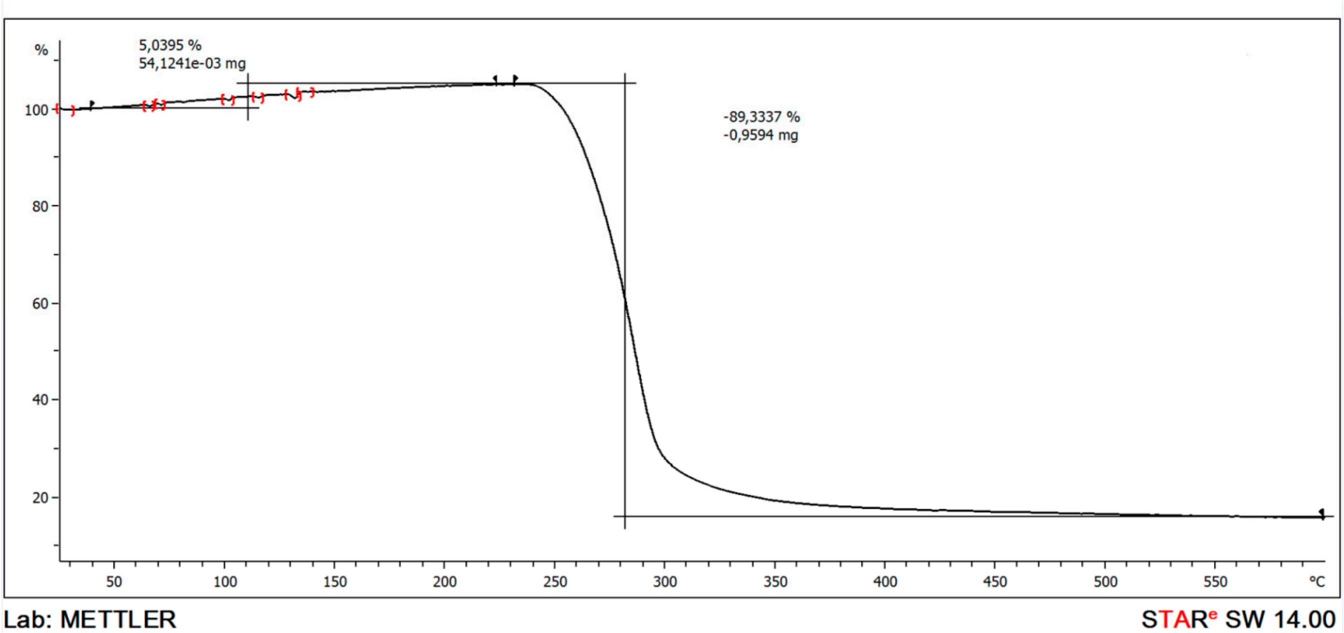

Figure S15. TGA of **3**

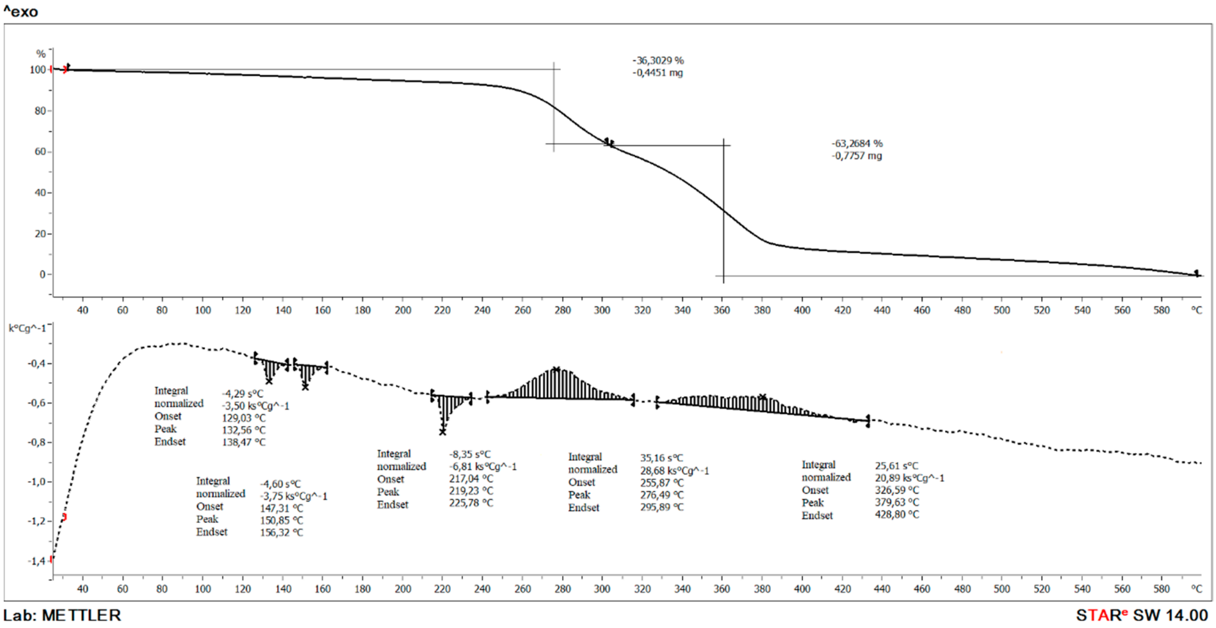

Figure S16. TGA and DSC of **4**
